# Curcumin in Food Technology: Trends, Challenges, and Innovative Perspectives Through Bibliometrics

**DOI:** 10.1002/fsn3.71903

**Published:** 2026-06-05

**Authors:** Vitória Damaceno Bueno, André Arigony Souto

**Affiliations:** ^1^ Graduate Program in Cellular and Molecular Biology Pontifical Catholic University of Rio Grande do Sul Porto Alegre RS Brazil; ^2^ School of Engineering Pontifical Catholic University of Rio Grande do Sul Porto Alegre RS Brazil

**Keywords:** bioactives, bioavailability, controlled release, curcuminoids, delivery systems, encapsulation, functional foods, nanotechnology, solubility, stability

## Abstract

Curcumin is a promising bioactive compound for functional foods, but its application remains limited by low solubility, poor oral bioavailability, and restricted stability in food systems. This study aimed to map the evolution of curcumin research and to identify the main technological strategies being developed to improve its use in food applications. A bibliometric analysis of Web of Science Core Collection records was combined with a focused review of food‐oriented delivery and formulation approaches. The results showed marked growth in scientific output, strong international collaboration, and a clear shift from pharmacological research toward technologies designed to improve stability, dispersibility, and bioaccessibility. Across both the main dataset and the focused bioavailability subset, nanotechnology, encapsulation, and delivery‐system design emerged as dominant themes, confirming that bioavailability is a central bottleneck guiding current research. However, the available evidence remains heterogeneous across in vitro, animal, human, and food‐matrix studies. Overall, curcumin remains a promising functional ingredient, but its broader industrial application will depend on scalable technologies, validation in real food matrices, and clearer regulatory pathways.

## Graphical abstract

This graphical abstract summarizes the bibliometric trends, technological challenges, and innovative applications of curcumin in food technology, highlighting its relevance in functional foods, encapsulation systems, bioavailability enhancement, and industrial translation.

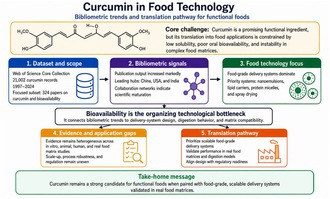

## 1 Introduction

*Curcuma longa* L., commonly known as turmeric, is a rhizomatous plant from the Zingiberaceae family, widely recognized for its traditional use in culinary, medicinal, and pharmaceutical applications. Native to Southeast Asia—particularly India, the world's leading producer and exporter—it is also cultivated in countries such as Bangladesh, China, Indonesia, and Peru (Prasad et al. 2014). Its rhizome, once dried and ground, yields a yellow‐orange powder extensively used as a spice, natural dye, and essential component of curry.

**FIGURE 1:**
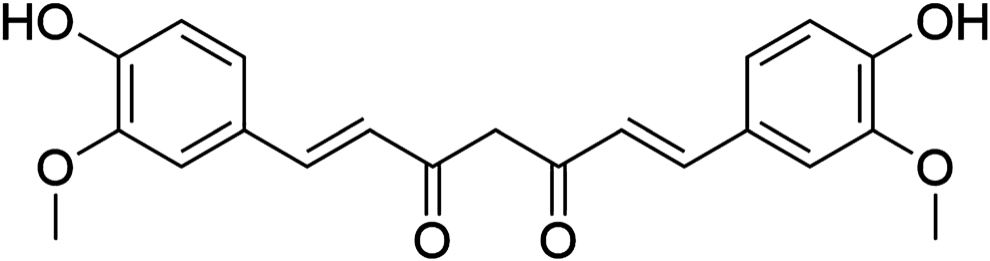
Chemical structure of curcumin, the main bioactive compound of *Curcuma longa* .

The primary phenolic compound in *Curcuma longa* is curcumin, which is responsible for its characteristic color and, more importantly, for its multiple pharmacological properties. Curcumin is a lipophilic polyphenol chemically known as diferuloylmethane [(1E,6E)‐1,7‐bis‐(4‐hydroxy‐3‐methoxyphenyl)‐1,6‐heptadiene‐3,5‐dione] (Figure 1). Its highly conjugated structure contributes to potent biological activities, including antioxidant, anti‐inflammatory, antimicrobial, neuroprotective, and anticancer effects (Jurenka 2009; Abrahams et al. 2019; Vallianou et al. 2015; Sarkar et al. 2016). Recent reviews also highlight the growing relevance of nanocarrier‐based strategies to overcome the physicochemical limitations of curcumin and improve its translational potential in food and pharmaceutical systems (Sun et al. 2012; Jacob et al. 2024; Tabanelli et al. 2021). Several clinical studies have highlighted its safety even at high doses (up to 12 g/day), administered over extended periods without significant adverse effects (Goel et al. 2008). Moreover, curcumin has been extensively investigated as a therapeutic agent in chronic noncommunicable diseases such as Alzheimer's (Goozee et al. 2015), type 2 diabetes (Nabavi et al. 2015), cardiovascular diseases (Jiang et al. 2017), and cancer (Mbese et al. 2019).

Given its well‐established bioactivity and the increasing demand for functional ingredients in the food industry, curcumin has become a topic of interest in the formulation of health‐oriented food products. In this context, the present work aims to provide a comprehensive literature review on the use of curcumin in food technology, covering the bibliometric landscape of scientific production as well as the key challenges and technological advances in its incorporation into food matrices. Through an integrative approach, this study seeks to map research trends, explore current barriers, and highlight innovative perspectives that could enable its functional application on an industrial scale.

## 2 Methodology

The present bibliometric analysis was conducted using the Web of Science—Core Collection (WoSCC) database, selected due to its rigorous indexing criteria, broad multidisciplinary coverage, and high‐quality metadata, characteristics that make it widely used in scientometric studies. The use of a single robust database, combined with consistent methodological criteria, is recognized as an appropriate approach for bibliometric analyses focused on identifying consolidated scientific trends (Donthu et al. 2021). No custom code or scripted pipeline was used in this study. Analyses were performed in VOSviewer (v1.6.20) and Microsoft Excel.

The search strategy employed the term “Curcumin” in the title field, covering the period from 1949 to October 2024. The title‐field restriction was maintained deliberately to prioritize records in which curcumin was the central object of investigation rather than a secondary analytical component. The exact search query applied in the Web of Science advanced search interface was TI = (“curcumin”). All indexes available within WoSCC (SCI‐Expanded, SSCI, ESCI, and others) were included, and no restrictions were applied regarding document type, language, or research area.

This strategy initially retrieved 21,264 records. However, documents published between 1949 and 1996 were found to present missing or inconsistent structured information, such as keywords and abstracts, which are essential for network, co‐occurrence, and thematic mapping analyses. Therefore, 262 records corresponding to this period were excluded, resulting in a final dataset of 21,002 documents published between 1997 and 2024 included in the analysis. This temporal delimitation ensured methodological consistency and reliability in both qualitative and quantitative analyses.

The data retrieved from WoSCC were exported in plain text format (.txt), containing all relevant bibliographic information, including titles, authors, affiliations, keywords, abstracts, and cited references. Records were exported using the “Full Record and Cited References” option, in batches when necessary due to WoS export limits, and subsequently merged into a single dataset prior to analysis. All data collections were performed within a consecutive 7‐day interval to ensure database consistency and minimize variations resulting from periodic platform updates.

Data analysis and visualization were performed using VOSviewer software, version 1.6.20, a tool widely applied in scientific mapping and network analysis of collaboration, term co‐occurrence, and co‐citation. VOSviewer is one of the most widely used tools for bibliometric visualization and science mapping, particularly for co‐occurrence and collaboration analyses (van Eck and Waltman 2009). The following parameters were applied in VOSviewer: Type of analysis: Co‐occurrence; Unit of analysis: All Keywords; Counting method: Full counting; Minimum number of occurrences per term: 5; Normalization method: Association strength; Layout: default VOSviewer layout; Clustering resolution: default (VOSviewer internal algorithm).

The methodology implemented in VOSviewer involved the construction of maps based on textual data, using title and abstract fields as sources for term extraction. The options to exclude structured abstract labels and copyright statements were activated to ensure data purity. Full counting was adopted as the counting method, and a minimum threshold of five occurrences was established for a term to be included in the analysis.

Keyword normalization and term extraction procedures were conducted using the standardized routines provided by VOSviewer. Additionally, a manual thesaurus file was applied to improve term consistency, including:

- Unification of singular and plural forms (e.g., “nanoparticle”/“nanoparticles”)
- Standardization of orthographic variations (e.g., “nano‐emulsion”/“nanoemulsion”)
- Grouping of semantically equivalent terms (e.g., “drug delivery”/“delivery system”)

This step ensured that conceptually identical terms were treated as a single node in the network analysis. Document types and language were maintained according to the default indexing of the Web of Science—Core Collection, without the application of additional filters to preserve the breadth and comparability of the analyzed records. The normalization of duplicate keywords considered orthographic variations, singular/plural forms, and semantically equivalent terms, following the automated procedures of VOSviewer, thereby ensuring consistency in the formation of thematic clusters. All data collections were performed within a consecutive 7‐day interval to ensure database consistency and minimize variations resulting from periodic platform updates.

The bibliometric analysis aimed to identify the annual number of publications in order to characterize publication growth, as well as to map the geographical distribution of studies and identify major knowledge‐production hubs. Co‐authorship networks were also examined to reveal collaborations among researchers and institutions. In line with the descriptive aim of the study, network interpretation combined cluster structure and total link strength with normalized output indicators (citations per document, c/doc), rather than formal graph‐theoretical centrality modeling. In addition, recurrent keyword analysis was conducted to detect emerging themes and priority research areas. Co‐citation analysis of references was employed to identify influential publications and conceptual landmarks in the development of curcumin research.

Complementarily, the exported records were also organized in Microsoft Excel, version 2410 (Build 16.0.18129.20158), to generate descriptive graphs, frequency tables, and comparative summaries. In response to reviewer concerns regarding thematic coherence, the complementary stage was refined into a targeted title‐field query combining the terms “curcumin” and “bioavailability.” The exact query used was TI = (“curcumin”) AND TI = (“bioavailability”). The full export contained 376 records, but to preserve temporal comparability with the primary dataset, only documents published up to 2024 were retained for descriptive synthesis (*n* = 324). A summary of the bibliometric workflow, dataset refinement process, and analytical use of each stage is presented in Table 1.

**TABLE 1:** Stage 1 retrieved 21,264 curcumin records from WoSCC, of which 262 pre‐1997 records were excluded due to sparse metadata, resulting in a primary analytical set of 21,002 publications (1997–2024). Stage 2 was refined using the combined curcumin + bioavailability title‐field strategy. Although the export contained 376 records, only publications up to 2024 were retained to ensure temporal comparability with Stage 1, resulting in a complementary focused subset of 324 documents.

Stage	Search focus	Search field/logic	Timespan	Records identified	Exclusion/scope note	Analytical use
Primary dataset	Curcumin	Title field	1949–Oct 2024	21,264	262 records from 1949 to 1996 excluded because of incomplete metadata for thematic mapping	21,002 documents (1997–2024) used for full bibliometric mapping
Secondary focused subset	Curcumin + bioavailability	Combined title‐field query	Export refined to 2024 comparison frame	376	52 records published after 2024 excluded to preserve temporal comparability with Stage 1	324 documents used as a focused curcumin‐specific bioavailability subset

This refined complementary subset was not used to map bioavailability as an autonomous field. Instead, it was used as a focused analytical lens on how curcumin‐specific studies operationalize absorption, pharmacokinetics, delivery systems, and formulation challenges. This adjustment strengthens the internal coherence of the manuscript by ensuring that the secondary bibliometric stage remains directly anchored to curcumin‐related literature.

The bibliometric analysis aimed to identify publication growth, major geographic hubs, collaboration structures, recurrent themes, and influential references in curcumin research. Co‐authorship, co‐occurrence, and co‐citation networks were therefore interpreted primarily as field‐mapping tools. In line with this descriptive objective, network interpretation combined cluster structure and total link strength with normalized output indicators (citations per document, c/doc), rather than formal graph‐theoretical centrality modeling. This choice was made because the study was designed to characterize broad thematic organization and collaboration patterns across a large interdisciplinary corpus, not to test topology‐dependent hypotheses about node influence or network hierarchy. Accordingly, metrics such as betweenness centrality, degree centrality, modularity, and density were considered outside the main analytical scope of the present integrative bibliometric review.

Flow summary of the bibliometric stages.

### 2.1 Methodological Considerations and Scope of the Study

Although the bibliometric approach adopted in this study enables the identification of global patterns and consolidated trends in curcumin research, certain methodological limitations inherent to the method and to the selected database must be explicitly acknowledged.

The exclusive use of the Web of Science—Core Collection may introduce selection bias, as this database prioritizes high‐impact journals, predominantly published in English, and subject to stringent indexing criteria. Consequently, relevant scientific outputs published in regional journals, gray literature, technical documents, patents, or publications not indexed in WoS may not be fully represented. This bias primarily affects the absolute quantitative assessment of scientific production and may also delay the visibility of recent studies because citation accumulation is time‐dependent.

Additionally, the choice of the term “Curcumin” in the title field, while enhancing the specificity and relevance of retrieved records, may result in the exclusion of studies in which curcumin is addressed as a secondary component or integrated within broader investigations. Thus, the results should be interpreted as a conservative mapping of research in which curcumin is the explicit central topic, rather than an exhaustive retrieval of every study mentioning curcumin only in abstracts or keywords.

Regarding keyword normalization, despite the use of standardized routines provided by VOSviewer, terminological and semantic variations are inherent to an interdisciplinary field such as food science and technology. Bibliometric results are therefore partly dependent on author keyword choices, indexing practices, and semantic proximity among terms. The identified thematic clusters represent dominant and recurrent groupings, but they do not exhaust all possible conceptual approaches associated with the topic.

Finally, the refined complementary bibliometric analysis should be interpreted as a targeted curcumin‐related subset rather than as a prevalence estimate of the broader bioavailability field. This distinction improves thematic specificity, but caution remains necessary when extrapolating evidence derived from pharmaceutical, animal, or highly controlled laboratory models to real food matrices and industrial conditions.

A further limitation is that the network analysis was intentionally descriptive and visualization‐oriented. Although graph‐theoretical indicators such as betweenness centrality, degree centrality, modularity, and density can provide additional topological detail, they were not used here because the purpose of the study was to map broad research trends and thematic organization rather than to perform a topology‐focused analysis of node influence. Future studies using narrower datasets or hypothesis‐driven scientometric designs may complement the present mapping by incorporating these advanced network metrics.

To estimate the potential exclusion bias associated with the conservative title‐field retrieval, a sensitivity analysis was conducted comparing the title‐only strategy with a broader title + abstract search. The title‐only search retrieved 20,959 records, whereas the title + abstract strategy retrieved 30,196 records, representing an increase of 9237 records. Thus, the title‐only approach excluded approximately 30.6% of the broader dataset, or, conversely, the broader strategy increased retrieval volume by about 44.1% relative to the title‐only corpus. Despite this numerical difference, the comparative bibliometric assessment showed that the principal patterns of the field remained stable, including the main thematic organization and leading publication hubs. This result suggests that the title‐only strategy primarily increases specificity rather than distorting the overall structure of the field. For this reason, the title‐based dataset was retained as the main analytical corpus, as it more conservatively represents studies in which curcumin is the explicit central topic.

Thus, the limitations discussed herein do not invalidate the presented results but delineate their interpretative scope, reinforcing the need for bibliometric findings to be analyzed in conjunction with experimental, regulatory, industrial, and food‐matrix evidence when discussing the technological development of curcumin in the food industry.

## 3 Results and Discussion

### 3.1 Bibliometric Analysis of Curcumin

#### 3.1.1 Keyword Analysis in 10‐Year Intervals

The analysis of keywords related to curcumin research, conducted in 10‐year intervals, allows the identification of structural shifts in scientific priorities and provides insight into how the field has evolved from exploratory investigations toward technological and translational approaches. Although publications in the Web of Science database date back to 1949, the older records lacked available keywords for mapping. From 1987 onward, the data became more consistent and suitable for structured interpretation.

Between 1987 and 1996, curcumin‐related studies were predominantly exploratory, focusing on biochemical characterization and general properties of the compound. Keywords such as “antioxidant,” “pharmacological properties,” and “turmeric” dominated the scientific literature, reflecting the initial interest in uncovering the biological potential of curcumin. From an analytical perspective, this pattern indicates that curcumin was investigated primarily as an isolated bioactive molecule, with limited articulation with technological applications or food systems, which is consistent with the early stage of research on natural polyphenols during that period (Aggarwal and Harikumar 2009).

Between 1997 and 2006, there was a significant expansion in the scope of research. Keywords like “anti‐inflammatory,” “neuroprotection,” “cancer,” and “preclinical models” signal a transition from chemical characterization to mechanistic and preclinical investigation. This period marks a transition toward targeted preclinical studies, especially involving therapeutic effects in animal models of diseases such as cancer and Alzheimer's disease. Curcumin began to consolidate as a promising therapeutic agent.

From 2007 to 2016, curcumin research was propelled by technological advances, particularly those addressing its low bioavailability. Keywords such as “nanotechnology,” “clinical trials,” “bioavailability,” and “inflammatory diseases” reflect a paradigm shift. The reorganization of keyword clusters explicitly reflects the recognition that low bioavailability constitutes the main bottleneck limiting the practical application of curcumin, directing scientific efforts toward the development of delivery systems capable of improving solubility, stability, and absorption. From a food technology perspective, this period represents the convergence of pharmaceutical and food research, with the incorporation of strategies such as nanoemulsions, liposomes, and encapsulation within edible matrices—an approach widely discussed in recent literature and considered promising for overcoming solubility, stability, and bioavailability limitations of curcumin in food systems (Kan et al. 2025; Pino 2026; Rahim 2025).

In the most recent period, from 2017 to 2024, research adopts a more translational and systemic approach, focusing on practical applications and a detailed understanding of curcumin's mechanisms of action. Keywords such as “gut microbiota,” “metabolic diseases,” “pharmaceutical formulation,” and “biomarkers” reflect a growing interest in exploring interactions between curcumin and complex biological systems. This pattern indicates that curcumin research has increasingly considered the interplay between food matrix, metabolism, gut microbiota, and physiological response, aligning with contemporary demands in functional nutrition and food innovation. Reviews published after 2022 confirm that the integration of delivery systems, food matrices, and metabolic effects represents one of the main research fronts involving curcumin, particularly focusing on encapsulation strategies applied to food preservation and bioavailability enhancement in complex matrices (Lan et al. 2023; Karaca et al. 2025; Buniowska‐Olejnik 2024).

Taken together, the temporal evolution of keywords demonstrates that the trajectory of curcumin research is guided by clearly identifiable technological bottlenecks, with bioavailability emerging as the central factor driving the transition from fundamental knowledge to technological applications. Unlike previous bibliometric analyses that are often limited to descriptive accounts of scientific production, the present results demonstrate how the emergence of specific technological themes has actively shaped the development of the field, thereby justifying the subsequent focus on formulation strategies and delivery systems discussed in the following sections. In this sense, bibliometrics is employed not merely as a descriptive tool, but as an analytical framework to understand the evolution and current priorities of curcumin research in food systems.

#### 3.1.2 Publications by Country Over 10‐Year Intervals

**FIGURE 2:**
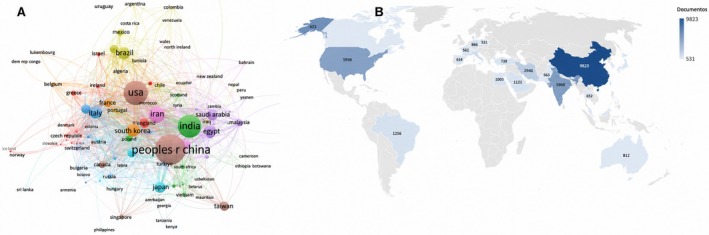
Global overview of curcumin research (1997–2024). (A) Simplified international collaboration network of the leading countries in curcumin research. Node size is proportional to document output, links indicate collaboration strength, and colors represent the main collaboration clusters. (B) Geographic distribution of publication volume. This figure should be interpreted together with the country‐level quantitative results reported in the text, including documents and citations per document (c/doc) because the map is intended to visualize collaboration structure rather than to provide a stand‐alone ranking of scientific influence.

The analysis of scientific production on curcumin across three 10‐year periods (1997–2006, 2007–2016, and 2017–2024) reveals a significant evolution in the geographical distribution of research (Figure 2), reflecting structural shifts in global scientific priorities and investment capacity in applied science and technology. In the initial period (1997–2006), the United States led in publications (403), followed by India (199) and Japan (94). China and South Korea began to gain relevance, while Brazil showed modest participation (13 publications). This pattern reflects the initial concentration of curcumin research in countries with well‐established traditions in biomedical and pharmaceutical research, where studies were mainly focused on elucidating biological mechanisms and therapeutic applications, with limited integration into food science.

In the subsequent decade (2007–2016), China assumed leadership (1612 publications), driven by strong investment in scientific research. The United States (1594) and India (1191) remained competitive, while South Korea (352) and Japan (300) consolidated their contributions. Brazil exhibited significant growth (139 publications), reflecting increased research funding and international collaborations.

China's rise during this period coincides with national policies targeting innovation in nanotechnology, functional materials, and bioactive ingredients—areas directly related to emerging strategies aimed at overcoming technological limitations of curcumin, such as low bioavailability. This shift also marks the growing engagement of food science with curcumin research, particularly in encapsulation and formulation systems, bringing scientific output closer to industrial applications.

Between 2017 and 2024, China further expanded its dominance (4074 publications), diversifying research into areas such as nanotechnology. India (1716) surpassed the United States (1456), reinforcing its scientific relevance. Brazil, with 545 publications, consolidated its position as a leader in Latin America, demonstrating greater international integration. Countries such as Iran (1710), Egypt (454), and South Korea (377) also emerged as significant contributors.

This scenario highlights the globalization of curcumin research and the increasing involvement of emerging countries, particularly those with strong traditions in the use of natural compounds and strategic interest in functional ingredients for foods and nutraceuticals. The more diversified geographical distribution suggests that curcumin has moved beyond basic or pharmaceutical research and has become strategically positioned within innovation agendas focused on food security, metabolic health, and functional food development.

From an analytical perspective, these results indicate that scientific leadership is not explained solely by publication volume, but by the convergence of funding policies, technological capacity, and industrial interest—factors that directly influence the types of technologies developed for curcumin application in food systems. This perspective helps explain why certain technological approaches, such as nanoemulsions and biopolymer‐based delivery systems, emerge more prominently in specific countries, a topic further explored in the subsequent sections.

#### 3.1.3 Analysis of Publications and Collaborations on Curcumin (1997–2024)

The bibliometric analysis of curcumin focused on the period from 1997 to 2024, although earlier publications exist in the Web of Science database. This temporal delimitation was adopted deliberately, as older records, while relevant, lacked complete bibliographic information, such as standardized keywords and structured abstracts. These limitations would hinder accurate term extraction and the construction of consistent thematic maps using VOSviewer.

From 1997 onward, substantial improvements in metadata standardization are observed, reflecting changes in editorial criteria and the consolidation of scientific indexing practices. This allows more robust, reproducible, and comparable bibliometric analyses over time. Based on this temporal framework, the results enabled the identification of consistent growth patterns in scientific production, the recognition of major research hubs, and the mapping of collaboration networks among countries and institutions.

These patterns indicate not only a quantitative increase in scientific output but also the formation of collaborative research ecosystems in which the development of delivery, formulation, and stabilization technologies for curcumin emerges as a central axis, aligned with contemporary demands in food science and industrial innovation. Recent studies confirm that this period coincides with intensified interdisciplinary collaboration among biomedical, pharmaceutical, and food science fields, reinforcing the relevance of curcumin as a functional ingredient rather than merely an isolated bioactive compound (Karaca et al. 2025; Lan et al. 2023).

#### 3.1.4 Distribution of Publications by Country

China clearly leads scientific production on curcumin, with 9823 publications and more than 273,000 citations, corresponding to approximately 27.8 citations per publication and highlighting both the volume and impact of its contributions. The United States ranks second (5936 publications), with a strong presence in international collaborations (total link strength: 2159), followed by India (5300 publications), whose long‐standing tradition in the medicinal and food use of *Curcuma longa* L. is widely recognized. The leadership of these countries reflects not only the scale of scientific production but also the convergence of national funding policies, technological infrastructure, and strategic interest in natural compounds with therapeutic and food applications, such as curcumin.

Brazil, with 545 publications, stands out as the main contributor in Latin America. In Europe, Italy, Germany, and the United Kingdom show relevant scientific output. Collaboration networks reveal strong interactions among major hubs—China, the United States, India, and Europe—facilitating global knowledge advancement. From a food technology perspective, this geographical configuration is particularly relevant, as these countries host consolidated research centers in nanotechnology, food science, and functional ingredient development. These hubs are characterized by the convergence of scientific expertise, technological infrastructure, and alignment with industrial demands, factors widely recognized as determinants of innovation in bioactive delivery systems, including curcumin (McClements 2020; Karaca et al. 2025; Buniowska‐Olejnik 2024).

Figure 2 complements this country‐level discussion by combining a simplified collaboration map (Panel A) with a global publication distribution view (Panel B). Panel A emphasizes the structure of international partnerships among the leading countries, whereas the accompanying quantitative discussion in the text reports document volume together with citations per document (c/doc), a normalized impact indicator included to avoid interpretation based exclusively on absolute publication counts.

China, despite its high publication volume, still presents opportunities for broader global integration. These findings reinforce that not only publication volume, but also the quality and extent of collaborative networks, are decisive for scientific advancement, an aspect recognized as critical for knowledge transfer and the development of technologies applicable to the food industry (Karaca et al. 2025).

#### 3.1.5 Co‐Authorship Analysis—Authors and Institutions

**FIGURE 3:**
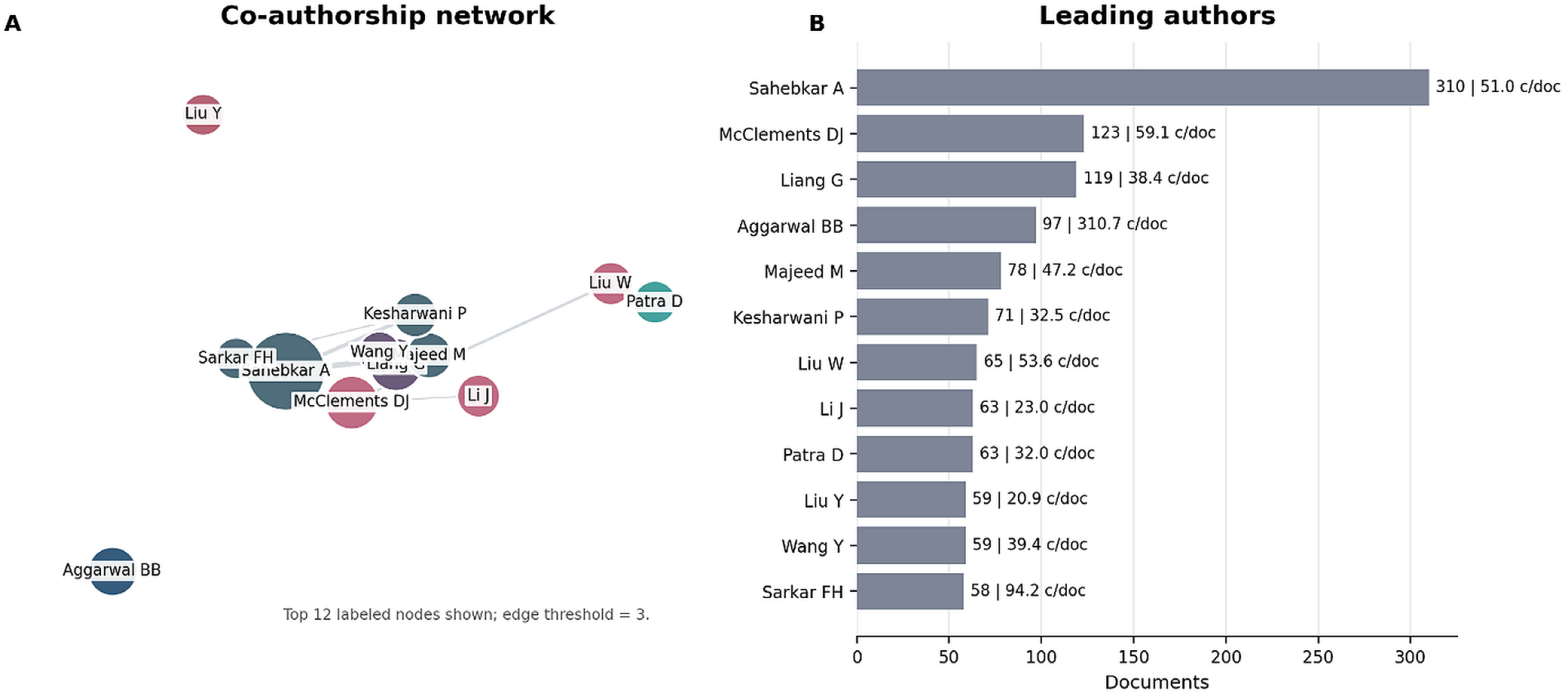
Co‐authorship network and leading authors in curcumin research. (A) Simplified network of the leading co‐authors. Node size is proportional to the number of documents, links indicate co‐authorship strength, and colors represent the main collaboration clusters. (B) Leading authors ranked by document output, with bar labels reporting citations per document (c/doc) as a complementary normalized impact indicator. The figure should be read as a combined structural and quantitative summary, in which network position visualizes collaboration patterns while c/doc helps contextualize normalized scientific impact.

Co‐authorship analysis identified the principal researchers driving curcumin research. Amirhossein Sahebkar led the dataset with 310 publications and approximately 51.0 citations per document, followed by David Julian McClements (123 publications; 59.1 c/doc) and Guang Liang (119 publications; 38.4 c/doc). Here, c/doc corresponds to total citations divided by the number of documents attributed to each author. These values are shown directly in Figure 3 to facilitate interpretation of the network map.

The presence of authors with diverse backgrounds—spanning pharmacology, biomedicine, and food science—demonstrates that curcumin research has evolved into a strongly interdisciplinary field, in which the integration of fundamental knowledge and technological development is essential to overcome limitations such as low bioavailability and instability in food systems. In particular, the central position of David Julian McClements within the co‐authorship network reinforces the role of food science and delivery systems as a structural axis of contemporary curcumin research, bridging pharmaceutical approaches with applications in edible matrices.

**FIGURE 4:**
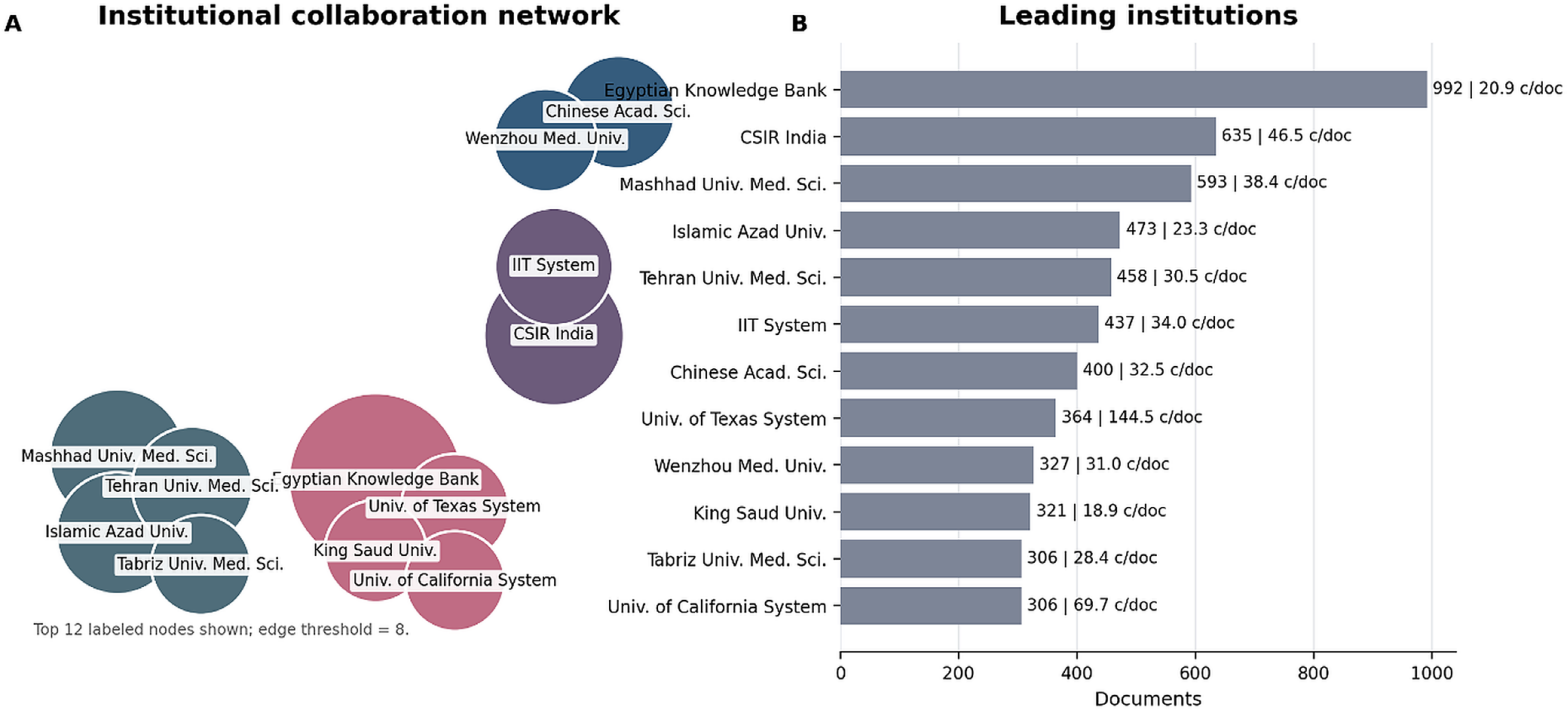
Institutional collaboration network and leading institutions in curcumin research. (A) Simplified collaboration map of the leading institutions. Node size is proportional to document output, links indicate collaboration strength, and colors represent the main institutional clusters. Only the most relevant labeled institutions above the visualization threshold are displayed to improve readability. (B) Leading institutions ranked by document output, with citations per document (c/doc) shown as a complementary impact indicator. The figure should be interpreted jointly with the quantitative values discussed in the text, because the map is intended to show collaboration structure rather than to function as an isolated measure of institutional influence.

At the institutional level, the ranking is headed by Egyptian Knowledge Bank (992 documents), followed by the Council of Scientific & Industrial Research in India (635 documents; 46.5 c/doc) and Mashhad University of Medical Sciences (593 documents; 38.4 c/doc). Panel A of Figure 4 shows the collaboration structure among the leading institutions, whereas Panel B provides the corresponding quantitative anchors.

Taken together, the author and institutional maps show that curcumin research depends on both prolific individual investigators and stable multi‐institutional collaboration structures. The author network is comparatively more specialized, while the institutional network is broader and more geographically distributed.

From an analytical standpoint, this distinction suggests that innovation in curcumin delivery technologies depends not only on highly productive individual researchers but also on institutional structures capable of sustaining long‐term, interdisciplinary collaborations—conditions recognized as essential for translating scientific findings into solutions applicable to the food industry (McClements 2020; Karaca et al. 2025). These bibliometric patterns directly guided the prioritization of the technological strategies discussed below, especially nanoemulsions, SLNs, protein‐based micelles, spray drying, and the related regulatory and industrial constraints.

#### 3.1.6 Keyword Co‐Occurrence Analysis

**FIGURE 5:**
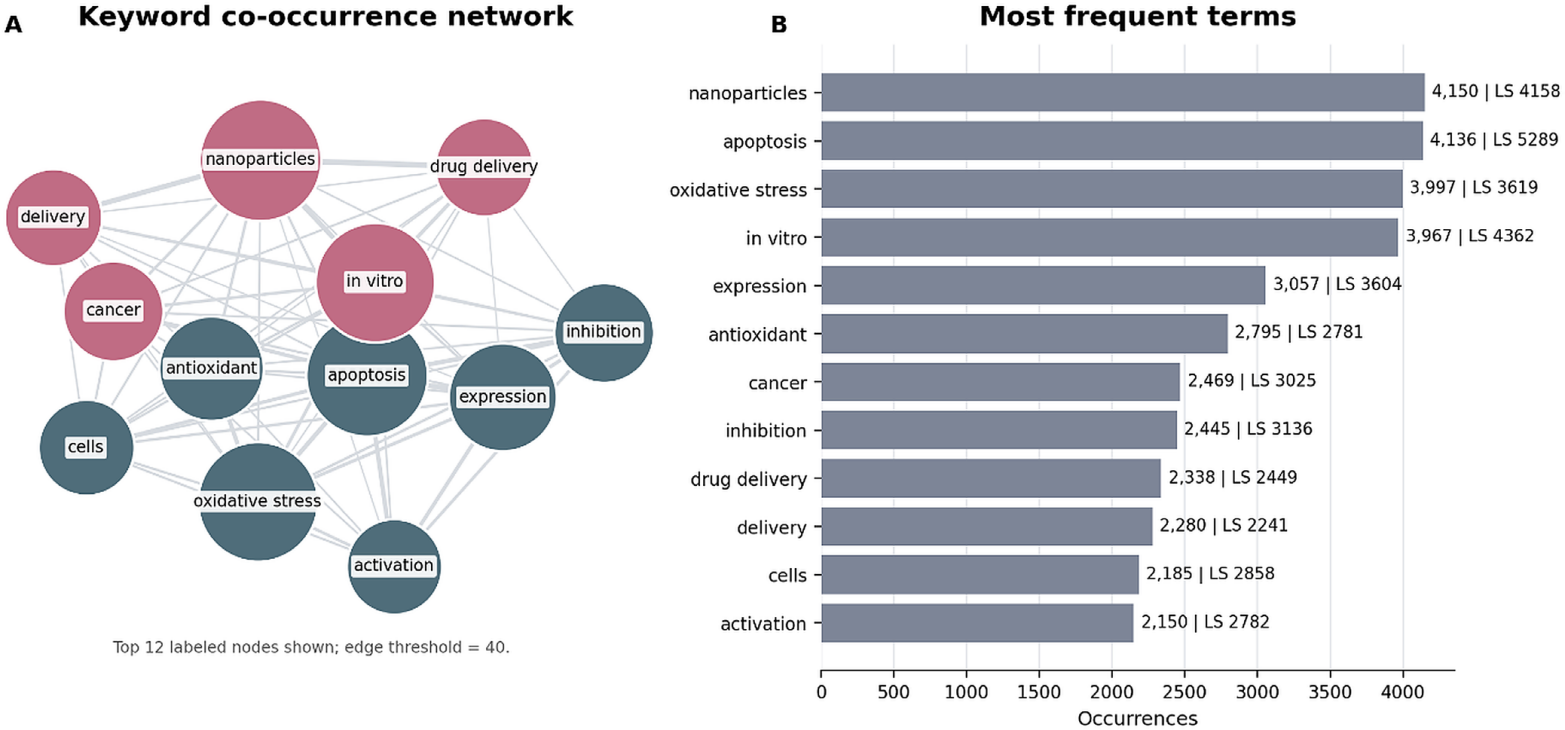
Keyword overview in curcumin research. (A) Simplified keyword co‐occurrence network restricted to the most visible labeled terms. Node size reflects keyword frequency, links indicate co‐occurrence strength, and colors represent the main thematic clusters. (B) Most frequent keywords in the main dataset. Bar labels report occurrences and total link strength (LS), where LS summarizes the cumulative co‐occurrence connectivity of each keyword. Terms combining high occurrence and high LS are both frequent and structurally integrated within the field. In this sense, the concentration of delivery‐related terms within the food‐relevant cluster provides the bibliometric basis for prioritizing nanoemulsions, lipid carriers, and related encapsulation strategies in the technological discussion.

Figure 5 combines a labeled keyword co‐occurrence network (Panel A) with a ranked summary of the most frequent terms (Panel B). After keyword normalization, the most recurrent terms in the main curcumin dataset are nanoparticles (4150 occurrences), apoptosis (4136), oxidative stress (3997), in vitro (3967), and expression (3057), indicating the strong prominence of mechanistic, cellular, and formulation‐oriented research. In this panel, the occurrence count expresses how often each term appears in the dataset, while the bar‐label parameter LS expresses how strongly each term is connected to the other displayed keywords.

In Panel B, LS denotes total link strength, that is, the cumulative co‐occurrence connectivity of each keyword with the other displayed terms. Terms with high occurrences but lower LS are frequent yet less integrated, whereas terms with both high occurrences and high LS occupy a more central position in the thematic structure. Panel A should therefore be read as a structural map of thematic proximity, while Panel B provides the quantitative anchors that make the dense network easier to interpret.

From a food technology perspective, the green cluster plays a strategic role, as it concentrates terms associated with the development of delivery systems compatible with edible matrices, such as nanoemulsions, lipid particles, and hybrid systems, evidencing the convergence between pharmaceutical and food research.

Bibliometric analysis revealed that although scientific production on curcumin has increased markedly over recent decades, particularly since 1997, a substantial proportion of studies remains focused on preclinical investigations and basic approaches, as indicated by the high frequency of terms such as “in vitro,” “oxidative stress,” and “apoptosis.” In parallel, the strong presence of keywords such as “nanoparticles,” “drug delivery,” and “bioavailability” demonstrates a clear recent shift toward addressing curcumin's biopharmaceutical limitations.

This pattern suggests that although technological strategies are well established at a conceptual level, their translation into food applications still faces challenges related to stability in complex matrices, process scalability, and regulatory acceptance.

Overall, despite widespread recognition of curcumin's therapeutic potential, low bioavailability remains one of the main challenges for both clinical and food applications. The growing interest in technological strategies aimed at improving bioavailability is reflected in the prominence of terms related to controlled release systems and nanotechnology. Consequently, a focused complementary bibliometric analysis restricted to curcumin‐related bioavailability studies is methodologically justified because it clarifies how this specific subset organizes around absorption, formulation, digestion behavior, and delivery design. This approach reduces descriptive fragmentation by explicitly connecting the observed bibliometric patterns to the technological and industrial discussion developed in the following sections, as recommended by recent studies in food science and bioactive delivery systems (McClements 2020; Karaca et al. 2025).

### 3.2 Targeted Complementary Analysis of Curcumin‐Related Bioavailability Studies

To directly address the reviewer's concern about thematic scope, the complementary bibliometric stage was redefined using a combined curcumin + bioavailability search. After restricting the export to publications up to 2024, the focused subset comprised 324 documents and 25,365 citations. Because every retained record explicitly links curcumin and bioavailability at the retrieval stage, this dataset offers a more coherent bridge between the broad curcumin mapping and the technology‐oriented discussion that follows.

Rather than treating bioavailability as an independent field, this focused subset shows how curcumin research specifically organizes around oral absorption, pharmacokinetics, delivery systems, digestion behavior, and formulation design. In this way, the secondary analysis supports interpretation of the main dataset without displacing it.

#### 3.2.1 Growth in Curcumin‐Bioavailability Publications Over Time

**FIGURE 6:**
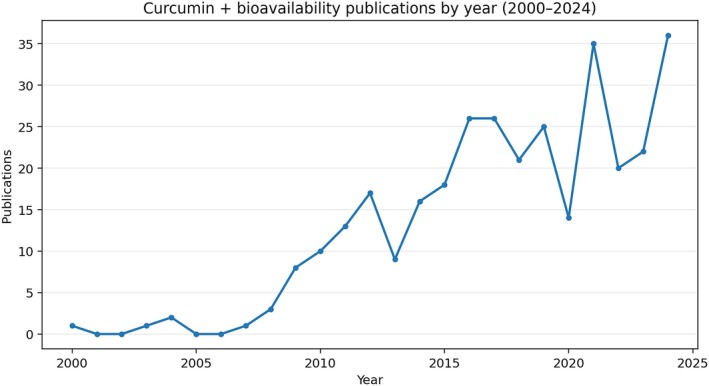
Annual number of publications retrieved in the targeted curcumin + bioavailability subset (2000–2024).

The targeted curcumin‐bioavailability subset begins in 2000 and shows sustained expansion over time (Figure 6). Annual output rose from isolated records in the early 2000s to 35 publications in 2021 and 36 in 2024, with the clearest acceleration after 2015. This pattern supports the interpretation of a maturing literature increasingly oriented toward formulation design, digestive behavior, and translational performance rather than only exploratory pharmacology.

This growth also reflects a qualitative shift in the literature. Earlier studies were more strongly centered on metabolism and systemic exposure, whereas more recent publications increasingly combine physicochemical stabilization, nanostructured delivery, and food‐compatible carrier design. Within the focused subset, original articles predominate (231/324; 71.3%), followed by reviews (43; 13.3%) and meeting abstracts (27; 8.3%), indicating that the field is driven mainly by experimental work but is already supported by a relevant body of synthetic literature.

From a food technology perspective, the trend is especially relevant because it reveals that bioavailability is not treated as a peripheral pharmacokinetic afterthought. Instead, it functions as a central design criterion that shapes how curcumin is encapsulated, dispersed, protected during digestion, and ultimately positioned for possible incorporation into complex matrices.

From a food technology perspective, this growth reflects a transition from purely pharmacological approaches to integrated strategies that simultaneously consider the food matrix, physicochemical stability, industrial processing, and biological efficacy.

#### 3.2.2 Publication Distribution by Country in the Targeted Subset

**FIGURE 7:**
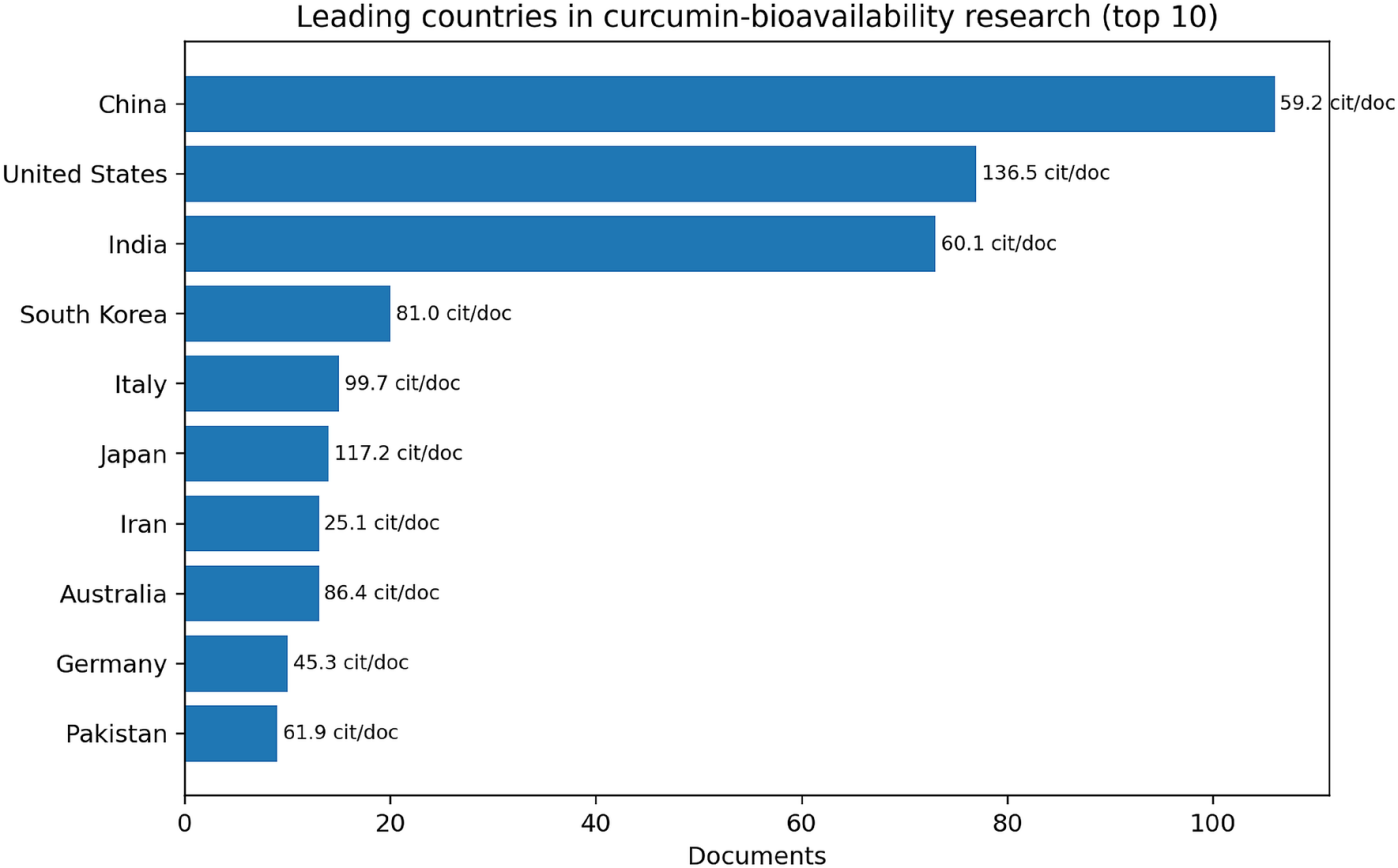
Leading countries in the targeted curcumin + bioavailability subset. Bars indicate publication volume; annotations show citations per publication. The figure should be interpreted through the combination of output and normalized impact, since leadership in this focused subset is not explained by volume alone but also by citation intensity and integration with formulation‐ and bioavailability‐oriented research.

The focused subset also reveals a more concentrated geographical pattern than that observed in the broader curcumin literature (Figure 7). China leads with 106 documents, followed by the United States (77) and India (73). However, when impact is normalized, the United States shows the highest citation intensity among the top three, with approximately 136.5 citations per publication, compared with 59.2 for China and 60.1 for India.

South Korea, Japan, Italy, and Australia appear with smaller publication volumes but strong citation density, indicating that leadership in this niche is not explained by volume alone. These differences suggest partially distinct research profiles: high‐volume formulation and delivery activity in Asian countries, alongside strong pharmacokinetic and translational influence from the United States and selected European groups.

Although countries such as Brazil appear with modest volume in this targeted subset, their presence confirms that curcumin‐bioavailability research has expanded beyond its historical core. Still, compared with the primary dataset, this niche remains more concentrated around specialized groups capable of integrating colloid science, pharmaceutical technology, and biological evaluation.

Analytically, this distribution reinforces that the bioavailability problem is most actively developed where formulation science, digestion studies, and translational testing converge. In other words, the geographic pattern supports the manuscript's broader argument that low bioavailability acts as an organizing technological bottleneck rather than a merely descriptive property of curcumin.

In this focused subset, secondary contributors are better interpreted through the combination of publication volume, citations per document, and collaboration strength than through raw output alone.

Comparison between the network map and the geographical map indicates that scientific progress in bioavailability is not determined solely by publication volume, but by the capacity to integrate into global collaborative networks—an essential factor for accelerating knowledge transfer to industrial and regulatory applications.

#### 3.2.3 Leading Authors and Institutions in the Targeted Subset

**FIGURE 8:**
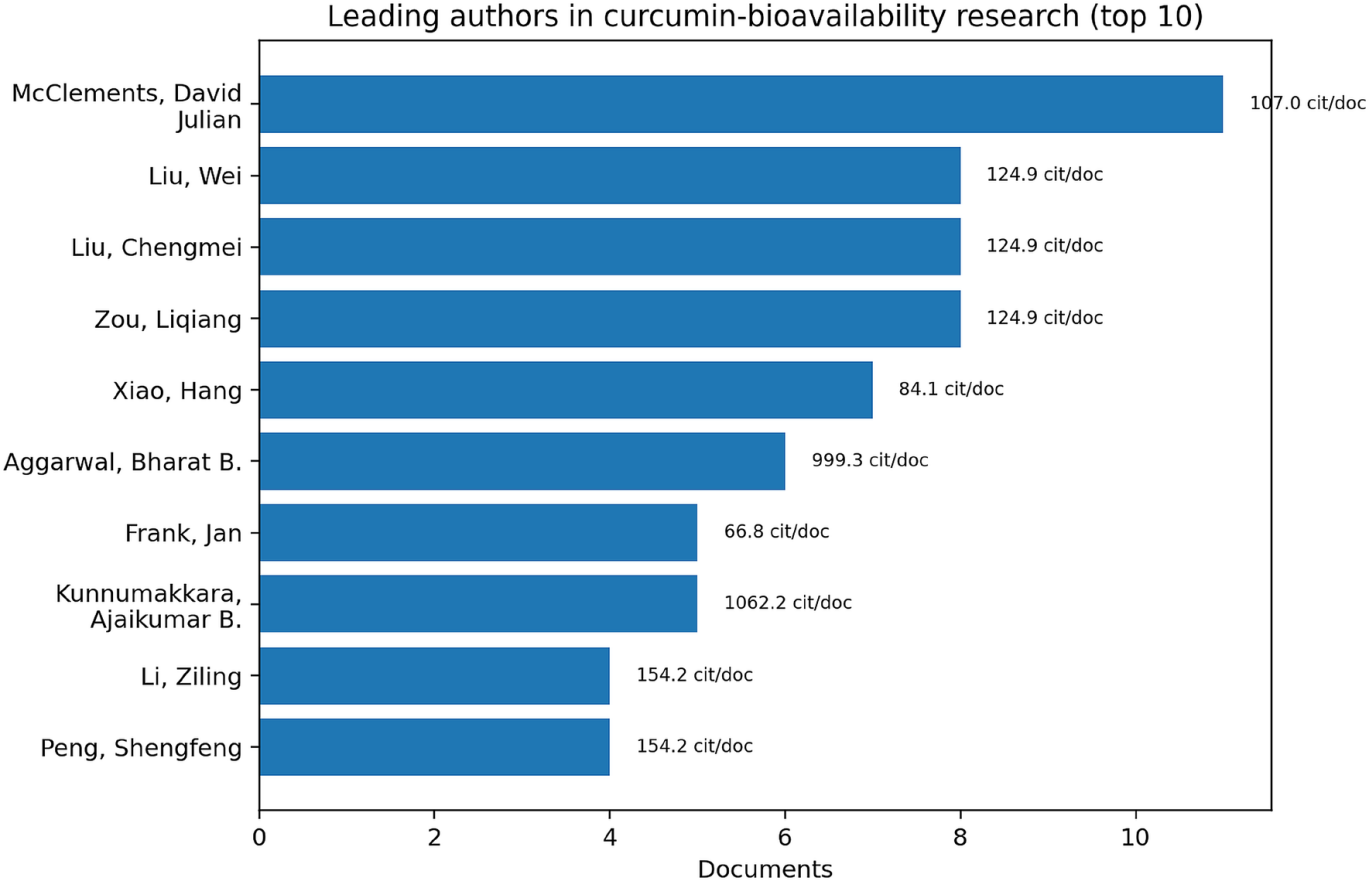
Leading authors in the targeted curcumin + bioavailability subset. Bars indicate publication volume; annotations show citations per publication. The figure highlights the coexistence of two complementary profiles in the field: highly productive formulation‐oriented authors and highly influential pharmacological contributors whose work continues to frame later food and delivery‐system studies.

At the author level, David Julian McClements leads the focused subset with 11 publications, followed by Wei Liu, Liqiang Zou, and Chengmei Liu with 8 publications each (Figure 8). McClements' prominence is especially meaningful for food technology because it reflects the central role of colloid science, emulsion design, and digestion‐oriented delivery systems in the effort to improve curcumin performance.

At the same time, highly cited contributors such as Bharat B. Aggarwal, Ajaikumar B. Kunnumakkara, and Preetha Anand illustrate another dimension of the field: seminal pharmacological and translational papers continue to shape how later food and formulation studies frame the bioavailability problem. The targeted subset therefore links foundational biomedical evidence with more recent carrier‐oriented strategies.

**FIGURE 9:**
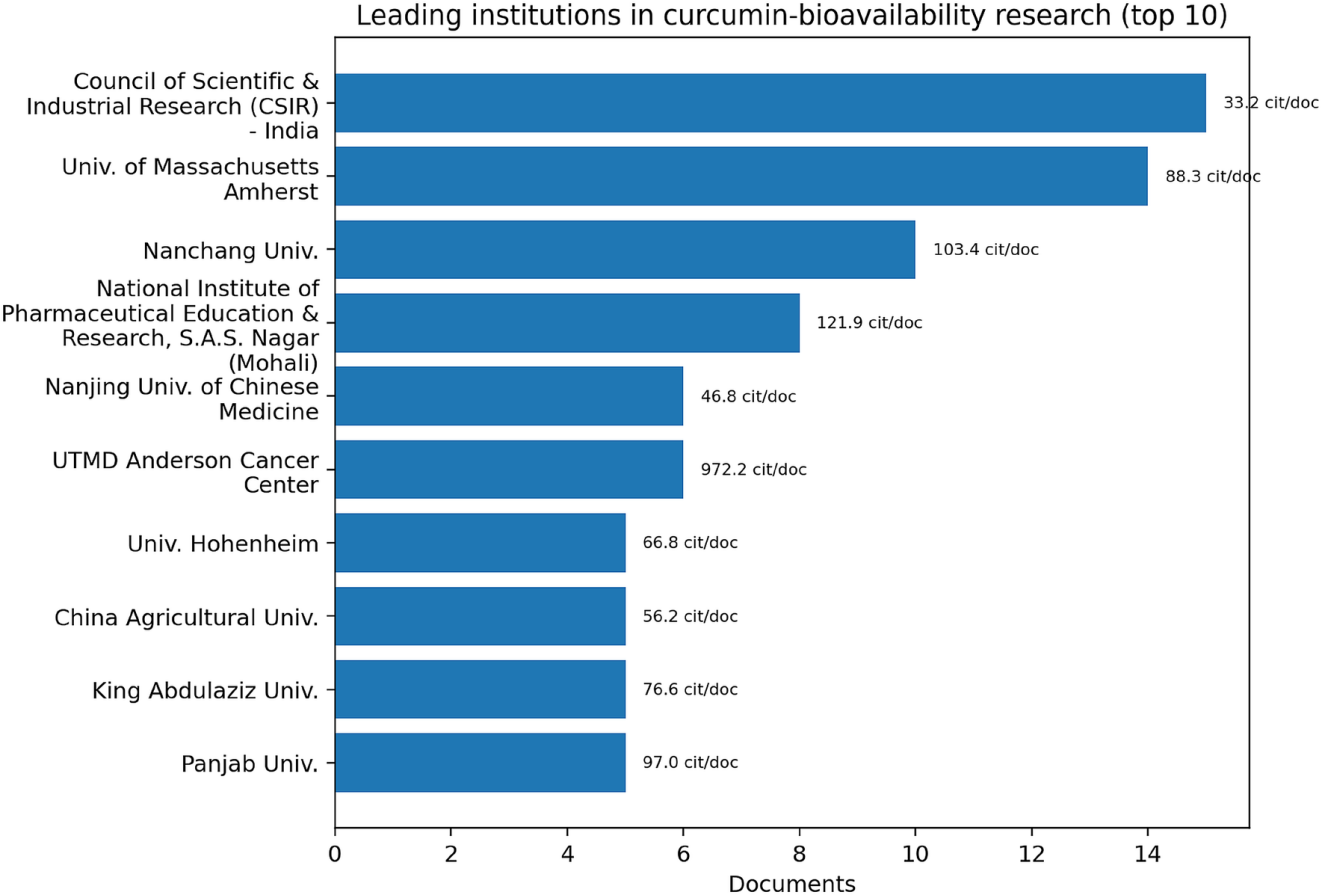
—Leading institutions in the targeted curcumin + bioavailability subset. Bars indicate publication volume; annotations show citations per publication. The figure should be interpreted as evidence that progress in curcumin bioavailability depends on interaction among food engineering, pharmaceutical sciences, and translational biomedicine, rather than on publication volume alone.

Institutionally, recurrent affiliations include the Council of Scientific & Industrial Research (India), the University of Massachusetts Amherst, Nanchang University, and the National Institute of Pharmaceutical Education and Research (India), while citation intensity is particularly high in institutions such as UT MD Anderson Cancer Center (Figure 9). This mix shows that progress in curcumin bioavailability depends on interaction among food engineering, pharmaceutical sciences, and translational biomedicine rather than on a single disciplinary axis.

Compared with the broader curcumin map, the targeted subset is more clearly structured around specialized centers able to connect formulation, analytical characterization, and biological evaluation. This concentration is consistent with a field that is moving from proof‐of‐concept claims toward more technically mature and application‐aware research designs.

Comparing the figures, collaboration among institutions appears broader and more structured than collaboration among individual authors, reinforcing the role of institutional alliances in advancing the field. This pattern indicates that larger‐scale projects—often supported by strategic funding and international partnerships—are decisive for technological maturation.

#### 3.2.4 Keyword Analysis in the Targeted Curcumin‐Bioavailability Subset

**FIGURE 10:**
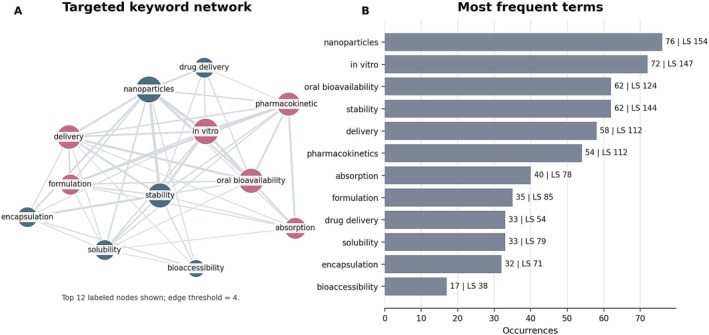
Targeted keyword overview in the curcumin + bioavailability subset after exclusion of the query terms. (A) Simplified co‐occurrence network of the principal labeled keywords. Node size reflects keyword frequency, links indicate co‐occurrence strength, and colors represent the main thematic clusters. (B) Most frequent terms in the focused dataset, with bar labels reporting occurrences and total link strength (LS), where LS summarizes the cumulative co‐occurrence connectivity of each term. Terms such as nanoparticles, stability, oral bioavailability, delivery, and pharmacokinetics, together with nanoemulsion, micelles, chitosan, and bioaccessibility, provide the quantitative and structural rationale for prioritizing nanoemulsions, SLNs, and protein‐based micelles in the subsequent technological discussion.

Keyword analysis further clarifies the internal structure of this focused literature (Figure 10). After exclusion of the query terms themselves, the most recurrent keywords are nanoparticles (76 occurrences), in vitro (72), stability (62), oral bioavailability (62), delivery (58), and pharmacokinetics (54). These terms show that the targeted subset is organized around formulation design, absorption‐related mechanisms, and performance evaluation.

Terms such as nanoparticles, nanoemulsion, micelles, chitosan, delivery systems, and bioaccessibility link the subset directly to food‐grade structuring strategies. In parallel, pharmacokinetics, absorption, and permeability preserve the connection with classical biopharmaceutical assessment. The literature therefore operates at the interface between mechanistic evaluation and applied formulation design.

The simultaneous prominence of stability, solubility, and bioaccessibility is particularly relevant for the food sector because it indicates that curcumin bioavailability research increasingly considers digestion behavior and matrix‐dependent release, not only systemic exposure. This distinction helps prevent overinterpretation of fold‐increase claims that originate outside realistic food conditions.

Overall, the targeted subset supports the conclusion that low bioavailability is not an external theme imported into curcumin research. It is an internal organizing problem that directs the choice of carriers, processing routes, and experimental models used to make curcumin more functional and more translatable.

As in Figure 5, LS in Figure 10 denotes total link strength. The figure should therefore be read as a combined structural and quantitative summary: Panel A highlights how the principal formulation‐ and pharmacokinetic‐related terms are interconnected, while Panel B reports the corresponding frequencies and LS values. In other words, occurrences indicate thematic frequency, whereas LS indicates how strongly each term is connected within the focused curcumin + bioavailability network.

### 3.3 Enhancing Curcumin Bioavailability: Technologies and Applications in the Food Industry

The technological discussion below is presented as a direct extension of the bibliometric structure of the field rather than as an independent narrative review. In the main dataset, the prominence of terms such as nanoparticles, drug delivery, and bioavailability indicates that nanostructured delivery is a central organizing axis of contemporary curcumin research. This interpretation is reinforced in the targeted curcumin + bioavailability subset, in which nanoparticles (76), stability (62), oral bioavailability (62), delivery (58), and pharmacokinetics (54) emerge as dominant and strongly connected terms, together with food‐oriented signals such as nanoemulsion, micelles, chitosan, and bioaccessibility. On this basis, nanoemulsions, SLNs, and protein‐based micelles are prioritized because they correspond directly to the dominant delivery‐ and formulation‐oriented clusters, whereas spray drying is additionally prioritized because it represents a more industrially mature route for translating stabilization and bioaccessibility goals into scalable food systems.

From a biopharmaceutical standpoint, curcumin exhibits low water solubility and limited systemic persistence after oral administration, which restricts its effectiveness as a bioactive compound. Seminal reviews indicate that technological strategies can significantly alter the biological performance of curcumin, but with substantial variability depending on the experimental model (e.g., in vitro, simulated digestion, animal, human) and the type of delivery system employed (Anand et al. 2007). For this reason, when discussing quantitative gains reported in the literature, it is essential to distinguish systemic bioavailability (pharmacokinetics) from bioaccessibility (the fraction potentially available after digestion) and to recognize that results obtained in pharmaceutical or preclinical contexts cannot be directly extrapolated to complex food matrices without additional validation.

Among the approaches most aligned with food technology, lipid nanoemulsions and other food‐grade lipid systems stand out, as they tend to improve curcumin dispersibility and its release during digestion, thereby enhancing bioaccessibility in simulated physiological environments. Recent reviews on lipid‐based delivery systems for curcumin highlight important advances in designing nanostructures compatible with food ingredients, while emphasizing that industrial translation depends on processing stability (temperature, ionic strength, pH), cost, and consumer acceptance (Karaca et al. 2025). Complementarily, reviews on nanoemulsions for delivering bioactive compounds in foods reinforce that system performance depends on interfacial composition and matrix interactions, which influence stability and release during digestion (Jafari et al. 2018). Recent reviews specifically focused on curcumin nanoemulsions further reinforce the importance of encapsulation technologies for improving dispersibility, digestion behavior, and bioaccessibility in food‐compatible systems (Jiang et al. 2020).

Several studies report marked increases in curcumin absorption and bioavailability parameters when associated with nanostructured systems, frequently in the range of 10‐ to 40‐fold relative to free curcumin. It is important to note, however, that these values are largely linked to pharmaceutical formulations, preclinical assays, and animal studies and do not necessarily reflect curcumin performance in real food matrices or human dietary exposure. For example, Dutta and Ikiki (2013) reported up to a 39‐fold increase in oral bioavailability of curcumin incorporated into solid lipid nanoparticles (SLNs) in an animal model, attributed to enhanced solubilization and protection against early metabolism.

Within solid lipid systems, studies based on pharmaceutical formulations have shown relevant results regarding encapsulation efficiency and stability. In SLN formulations prepared by emulsification followed by cooling, encapsulation efficiencies above 80% have been reported, along with physicochemical stability maintained for up to 12 months at 5°C (Tang 2020). In vivo assays indicated that incorporating curcumin into SLNs resulted in an approximately 20‐fold increase in plasma concentration after oral administration in animal models (Malaekeh‐Nikouei and Salarbashi 2018). Although these findings demonstrate the high potential of nanostructured lipid systems, direct translation to foods requires caution, since such studies do not account for the complexity of food matrices or real industrial processing conditions.

Polymeric micelles have also been investigated as promising carriers for curcumin, especially when formulated from whey proteins and casein—materials widely accepted in the food industry. In vitro and cellular studies indicate that these micelles increase curcumin dispersibility and promote cellular uptake, suggesting potential applications in products such as fortified yogurts and protein shakes (Malaekeh‐Nikouei and Salarbashi 2018). However, most evidence is still based on simplified models, and additional evaluations in real foods and simulated digestion studies are required. Studies using whey‐protein complex microparticles also demonstrated improvements in curcumin bioaccessibility and controlled release during digestion, reinforcing the potential of protein‐based carriers in food systems (Ye et al. 2021).

Beyond nutritional applications, microencapsulation strategies for curcumin have also been explored in active food packaging, expanding the technological scope of this compound. Comprehensive reviews also highlight that spray drying, liposomes, cyclodextrin inclusion complexes, and electrospinning are among the most promising encapsulation approaches for food applications involving plant bioactives and curcumin stabilization (Cristina Muñoz‐Shugulí et al. 2021; Rashwan et al. 2022). In a study published in the International Journal of Food Science & Technology, Bian et al. (2023) developed an antioxidant paper containing curcumin microcapsules that exhibited antioxidant activity above 93% and significantly reduced oxygen and water vapor permeability. This approach extended cake shelf life, demonstrating the industrial applicability of encapsulated curcumin in non‐ingestible systems that are nonetheless relevant for food preservation.

Despite technological advances, wide variability in curcumin content in commercial raw materials remains a limiting factor. Quantification of curcumin in commercial *Curcuma longa* samples reveals substantial variation (0.06% to 2.06% w/w), reinforcing the need for rigorous quality control, raw material standardization, and traceability to ensure consistent technological performance and bioactivity (Messa et al. 2025). These aspects are particularly critical for the food industry, where reproducibility and regulatory compliance are decisive for the feasibility of functional products.

In parallel with the advancement of nanostructures, bibliometric data also show strengthening of approaches with higher industrial readiness, especially microencapsulation. In this context, techniques such as spray drying are particularly relevant because they are already widely used at industrial scale for sensitive compounds. Studies involving real food matrices demonstrate that spray‐dried microencapsulation of curcumin enables its incorporation into foods with consistent gains in stability and retention during processing and storage. A direct example is milk fortification using spray‐dried microencapsulated curcumin, in which characterization parameters and ingredient performance within the dairy system are discussed (Patel et al. 2021).

Additionally, comparisons between spray drying and freeze‐drying highlight performance differences related to bioactive retention and encapsulated material stability, providing a technical basis for selecting the method according to product type and processing conditions (Guo et al. 2022). In classic food encapsulation matrices (maltodextrin, gum arabic, and modified starch), it has also been shown that curcumin stability varies with carrier formulation and drying method, reinforcing the need for application‐specific optimization for each industrial use (Cano‐Higuita et al. 2015).

Multiple studies—particularly in pharmaceutical and preclinical contexts—report substantial increases in pharmacokinetic parameters of curcumin when associated with nanostructured systems. However, these values do not automatically represent performance in foods, because they often rely on animal models or non‐food formulations, frequently refer to systemic bioavailability, and because food matrices and processing can radically alter curcumin stability and release.

Therefore, within the food industry context, the most defensible and applicable interpretation is to view these technologies primarily as strategies to improve stability, dispersibility, and bioaccessibility, prioritizing evidence generated in real matrices and using industrially feasible processes. In this sense, integrating bibliometric findings with the technological review supports the view that the field is moving from a predominantly preclinical emphasis toward solutions increasingly aligned with functional food production, although significant gaps in industrial‐scale validation and real consumption conditions remain (Karaca et al. 2025; Patel et al. 2021).

Based on this interpretation, Table 2 was designed not merely as a qualitative summary of delivery systems, but as a structured comparative framework linking the principal technologies to their predominant evidence type, representative quantitative indicators, food‐application relevance, and main interpretive limitations. This format makes explicit that the largest fold‐increase claims in the literature are concentrated in pharmaceutical or animal‐based systems, whereas the strongest food‐sector evidence more often concerns stability, dispersibility, retention, and bioaccessibility in real matrices or industrially relevant processes. The table should therefore be interpreted as a comparative guide to technological positioning rather than as a direct ranking of superiority, since the reported indicators derive from different endpoints and experimental models.

**TABLE 2:** Main technologies for improving curcumin bioavailability/bioaccessibility, predominant evidence type, and level of applicability in the food industry.

Technology	System type	Predominant experimental model	Reported effect, food/industrial application and translation limitations	References
	Food	Simulated digestion, in vitro	↑ Bioaccessibility; high compatibility with beverages and dairy; stability depends on matrix	Jafari et al. (2018), Karaca et al. (2025)
SLNs	Pharmaceutical	Animal		
SLNs	Pharmaceutical	Animal	Encapsulation > 80%; ↑ plasma concentration ~20×; exploratory food application	Malaekeh‐Nikouei and Salarbashi (2018)
Protein micelles	Food	In vitro, cellular	↑ Dispersibility; potential for yogurts and shakes; limited real‐matrix studies	Malaekeh‐Nikouei and Salarbashi (2018)
Spray drying	Food	Food matrix	↑ Stability; established industrial technique; depends on carrier selection	Patel et al. (2021)
Freeze drying	Food	In vitro	↑ Bioactive retention; high cost and low scalability	Guo et al. (2022)
Polymeric microencapsulation	Food	Stability studies	↑ Thermal/oxidative stability; performance depends on formulation	Cano‐Higuita et al. (2015)
Active packaging	Food	Industrial	Antioxidant activity > 93%; shelf‐life extension; non‐ingestible	Bian et al. (2023)

Representative quantitative indicators are not methodologically equivalent across systems because they derive from different endpoints, including systemic bioavailability, plasma exposure, encapsulation efficiency, storage stability, antioxidant activity, and digestion‐related bioaccessibility. Accordingly, the table is intended as a structured comparative guide rather than as a direct ranking of technological superiority. Most large fold‐increase claims are derived from preclinical or pharmaceutical models and should not be directly extrapolated to food systems.

Taken together, the comparison in Table 2 reinforces that technological maturity in food applications is not determined solely by the magnitude of reported pharmacokinetic gains, but by the balance among functional performance, evidence type, matrix compatibility, process scalability, and regulatory feasibility. A structured summary linking bibliometric evidence to technology prioritization is presented in Table 3.

**TABLE 3:** Bibliometric signals supporting technology prioritization.

Bibliometric signal	Quantitative/structural evidence	Technology prioritized
Nanostructured delivery dominates the field	Nanoparticles = 4150 in main dataset; nanoparticles = 76 in targeted subset; strong LS and cluster integration	Nanoemulsions, SLNs
Bioavailability/stability as structuring bottleneck	Oral bioavailability = 62; stability = 62; delivery = 58; pharmacokinetics = 54	SLNs, micelles, nanoemulsions
Food‐compatible carrier design	Terms such as nanoemulsion, micelles, chitosan, bioaccessibility linked to food‐grade strategies	Protein‐based micelles, nanoemulsions
Industrial translation emphasis	Recurrent concern with stability, real matrices, and process scalability in technological review	Spray drying

Large fold‐increase claims should be interpreted cautiously, as they are derived mainly from pharmaceutical or preclinical models and do not constitute equivalent evidence of performance in real food systems. For food applications, the most relevant criteria are stability, dispersibility, bioaccessibility, matrix compatibility, and scalability.

## 4 Regulatory, Economic, and Industrial‐Readiness Challenges of Curcumin‐Based Technologies

Although scientific literature shows significant advances in technologies designed to improve curcumin stability and bioavailability, translation into industrial food applications remains uneven. The main barriers are not only technical but also regulatory, economic, and market‐related, particularly when formulation strategies alter exposure profile, processing requirements, or consumer perception (Muthu et al. 2025; Kamboj et al. 2026). The broader application of nanotechnology in foods has expanded considerably in recent years, particularly in delivery, preservation, and functional ingredient stabilization systems, although concerns regarding regulation, toxicity assessment, and consumer acceptance remain relevant challenges (He et al. 2016; Shafiq et al. 2020; Sahoo et al. 2022).

Regulatory positioning is clearer for conventional curcumin and turmeric forms than for advanced delivery systems. Curcumin is already authorized as food color E100 in the European Union and is regulated as a color additive by the U.S. Food and Drug Administration. In addition, EFSA re‐evaluations have reinforced the importance of exposure assessment and safety monitoring for curcumin use as food additive E100 in European food systems (EFSA 2020). In Brazil, curcumin and turmeric‐derived ingredients are regulated within the framework of food additives and supplements (European Commission 2026; FDA 2026a, 2026b; ANVISA 2018a, 2018b).

However, when formulation changes involve nanoencapsulation, altered particle size, or delivery systems explicitly designed to enhance bioavailability, the regulatory pathway becomes less straightforward. According to FDA guidance, changes in particle size, delivery mechanism, or bioavailability may require case‐by‐case safety reassessment, even when the original substance is considered safe (FDA 2014, 2022). Similarly, EFSA requires specific risk assessment when nanotechnology or small particles are involved, and in Brazil such systems may fall within the scope of novel foods or new ingredients, requiring additional evaluation (EFSA 2018, 2021; ANVISA 2021).

Concrete examples of more advanced translation include the incorporation of spray‐dried microencapsulated curcumin into milk and powdered food systems, demonstrating improved stability during processing and storage (Patel et al. 2021), as well as the use of curcumin microcapsules in active food packaging, where antioxidant activity above 93% contributed to shelf‐life extension (Bian et al. 2023). These examples illustrate applications that have moved beyond proof‐of‐concept and show why technologies combining scalability, formulation stability, and regulatory compatibility are more likely to approach practical implementation.

Using the TRL framework as an indicative interpretive lens rather than a formal certification, spray‐drying‐based systems appear closer to later‐stage translation because they rely on processing operations already established in the food industry. Selected food‐grade nanoemulsions can also be interpreted as relatively advanced in translational terms, although their regulatory pathway may still depend on formulation‐specific features. By contrast, SLNs and polymeric micelles remain better characterized as lower‐to‐intermediate readiness technologies since their evidence base is still more strongly concentrated in laboratory, preclinical, or simplified‐model conditions.

Overall, industrial translation depends less on the largest reported pharmacokinetic gain and more on whether a system can combine functional performance with food‐grade ingredients, process scalability, acceptable cost, regulatory feasibility, and consumer acceptance. From this perspective, spray‐dried systems and selected nanoemulsions currently appear closer to food‐sector implementation than more complex nanostructures whose technological promise remains ahead of routine industrial deployment.

## 5 Conclusion

This study shows that contemporary curcumin research is increasingly organized around formulation, delivery, and processing strategies aimed at overcoming low bioavailability. Bibliometric mapping revealed a large, collaborative, and technologically evolving field, while the focused curcumin + bioavailability subset confirmed that absorption, stability, and delivery remain its main organizing themes.

At the same time, not all reported advances have the same relevance for food applications. The largest pharmacokinetic gains are often associated with pharmaceutical or animal‐based systems, whereas food‐compatible technologies more consistently support improvements in stability, dispersibility, bioaccessibility, and matrix compatibility. Accordingly, spray drying, selected nanoemulsions, and protein‐compatible carriers currently appear more realistic for food‐sector translation than more complex systems that remain constrained by scale‐up and regulatory uncertainty.

Future progress will depend on validation in real food matrices, a clearer distinction between bioaccessibility and systemic bioavailability, and closer alignment between technological performance, industrial feasibility, and regulatory requirements. Under these conditions, curcumin can be more realistically positioned not only as a promising bioactive compound, but as a candidate ingredient for safe, scalable, and consumer‐accepted functional foods.

## Author Contributions

**André Arigony Souto:** writing – review and editing, project administration, supervision. **Vitória Damaceno Bueno:** investigation, writing – review and editing, methodology, data curation.

## Funding

This work was conducted with the support of the Coordination for the Improvement of Higher Education Personnel—Brazil (CAPES)—Funding Code 001.

## Conflicts of Interest

The authors declare no conflicts of interest.

## Data Availability

The datasets generated and analyzed during the current study, including the raw Web of Science Core Collection exports, merged bibliographic files, VOSviewer map files (.map and .net), and thesaurus‐based keyword normalization files used for preprocessing, are available from the corresponding author upon reasonable request. No custom code was used in this study; analyses were performed using VOSviewer (version 1.6.20) and Microsoft Excel.
